# Atretic Parietal Cephalocele With First Trimester Chiari Malformation and Sinus Pericranii Companion Case

**DOI:** 10.7759/cureus.21604

**Published:** 2022-01-25

**Authors:** Myra Ahmad, Hamzah Ahmad, Atif Ahmed, Jeffery R Blair, Eric J Basile, Salman Ahmad, Patricia E Roche

**Affiliations:** 1 Radiology, Touro College of Osteopathic Medicine, New York, USA; 2 Neurology, Touro College of Osteopathic Medicine, New York, USA; 3 Physical Medicine and Rehabilitation, Nova Southeastern University Dr. Kiran C. Patel College of Osteopathic Medicine, Davie, USA; 4 Internal Medicine, Idaho College of Osteopathic Medicine, Meridian, USA; 5 Internal Medicine, Touro College of Osteopathic Medicine, New York, USA; 6 Orthopedic Surgery, Touro College of Osteopathic Medicine, New York, USA; 7 Radiology, Stony Brook University, Stony Brook, USA

**Keywords:** neuroradiology, parietal encephalocele, chiari ii malformation, chiari i malformation, vascular neurology

## Abstract

Encephaloceles are the type of dysraphism in which a skull defect allows for herniation of meninges, with or without the inclusion of neural tissue, and are commonly associated with agenesis of the corpus callosum. Encephaloceles are classified as frontal, occipital, or parietal, with parietal cephaloceles, or vertex cephaloceles (VC), being the least common. Despite this, VCs present as the most common cause of a midline scalp mass, displaying complex venous and neural malformations commonly referred to as the “tip of the iceberg.” Atretic parietal encephaloceles (APC), a type of VC, are benign lesions arising from meningeal and vestigial tissue which have undergone fibrotic degeneration. As a result, prognosis will generally be better than other encephaloceles due to vestigial tissue involvement. Here, we report a neonate presenting with APC, corpus callosum agenesis, and a cingulate gyrus lesion, along with a sinus pericranii companion case for comparison.

## Introduction

Birth defects affect nearly 3% of all neonates [1]. While this may appear as a small number, birth defects account for nearly 20% of all infant deaths as the leading cause. For all birth defects, roughly 2% of live births are affected by central nervous system (CNS) defects, such as spina bifida (SB), anencephaly, or encephalocele, with encephalocele occurring every 1 in 10,000 live births [2].

Encephaloceles have been described as a form of dysraphism in which a skull defect allows for herniation of meninges, with or without the inclusion of neural tissue, and are commonly associated with agenesis of the corpus callosum [3]. Although etiology has been poorly understood [3,4], encephaloceles are thought to be a post-neurulation defect, as opposed to primary or secondary neurulation defects, as neural tissue, meninges, and spinal canal have completely formed [4].

Encephaloceles are classified as frontal, occipital, or parietal, with each having further sub-distinction. Parietal cephaloceles, or vertex cephaloceles (VCs), account for 15-38% of all cephaloceles, making them the least common of all cephaloceles [5,6]. Despite being the least common location for encephaloceles, VCs present as the most common cause of a midline scalp mass, yet display complex venous and neural malformations, commonly referred to as the “tip of the iceberg” [7,8].

Atretic parietal encephaloceles (APCs), found in 31% of all parietal encephaloceles, are benign lesions arising from meningeal and vestigial tissues which have undergone fibrotic degeneration [6]. As a result, prognosis will generally be better than other encephaloceles due to vestigial tissue involvement [8]. Here, we report a neonate presenting with APC, corpus callosum agenesis, and a cingulate gyrus lesion.

## Case presentation

A 22-hour-old female infant presented with a suspected encephalocele to the neonatal intensive care unit. The patient’s mother was a 39-year-old female with a past medical history significant for melanoma, status post excision with positive margins, and sentinel lymph node biopsy during the pregnancy. The mother was negative for typical congenital infections apart from unknown status of rubella and varicella. Five months prior to birth, a 7 mm posterior occipital encephalocele, along with a previously described Chiari malformation in the posterior fossa was noted on ultrasound. A subsequent fetal MRI was performed - a 3 mm T2-hyperintense lesion along the parietal scalp with no intracranial extension was noted and the Chiari malformation was not noted.

The mother presented to labor and delivery for a scheduled cesarean section. Artificial rupture of membranes one minute prior to delivery with clear fluid was noted. The delivery course proceeded without complications. The neonate posted appearance, pulse, grimace, activity, and respiration (APGAR) scores of 9/9, at one minute and five minutes. The newborn’s head was wrapped in plastic wrap at site of protrusion on posterior scalp to keep the area moist. The patient was put on 10% dextrose IV, and oral sucrose. The patient’s total fluid intake was 58.8 mL and output was 45 mL.

The patients’ vitals were stable, and physical examination showed the infant was awake in the incubator in no acute distress. A 1 cm protrusion was noted on the posterior aspect of the crown of the head with no breaks in the skin. A small indentation in the skull could be palpated immediately anterior to the protrusion, which was soft but full to palpation with no erythema. The remainder of the physical examination was normal.

Lab results were normal with exceptions of hemoglobin, elevated at 20.5 g/dL, white blood cell count at 18.33 x 10^9^/L, and platelets were greater than 164 x 10^9^/L. On non-contrast CT and MRI, an atretic parietal cephalocele, as well as dysplasia involving the corpus callosum and both cingulate gyri, was seen. Magnetic resonance angiography was within normal limits. Magnetic resonance venography showed findings consistent with atretic parietal cephalocele, with no thrombosis. No surgery was planned. The patient was to be discharged when tolerating full feeds with a stable temperature in an open crib. Follow-up with neurosurgery to monitor the encephalocele and ventricular size were recommended. The parents were advised to avoid trauma to the scalp and to protect the region.

**Figure 1:**
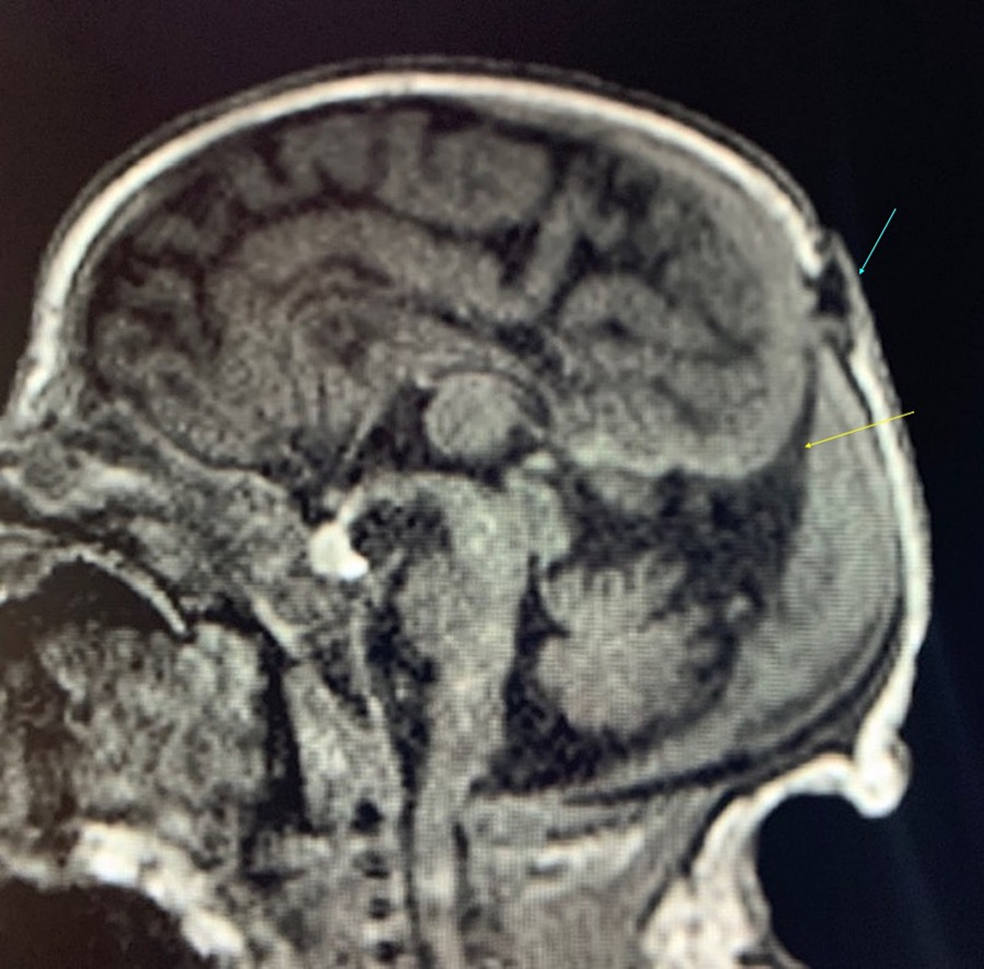
Sagittal FLAIR pre-contrast MRI. The image is showing superior beaking of the tentorium (yellow arrow) as it approaches the scalp lesion (blue arrow). FLAIR: fluid-attenuated inversion recovery

An MRI performed in the hospital with and without IV gadolinium contrast revealed a small cystic-appearing structure within the parietal scalp just at the midline, adjacent to the posterior fontanelle. The cyst measured approximately 0.7 x 0.4 x 1.0 cm in size. A cerebrospinal fluid (CSF) tract connecting this cystic structure with the intracranial CSF space, along with a primitive falcine vein and superior beaking of the posterior tentorium was seen (Figure 1).

**Figure 2:**
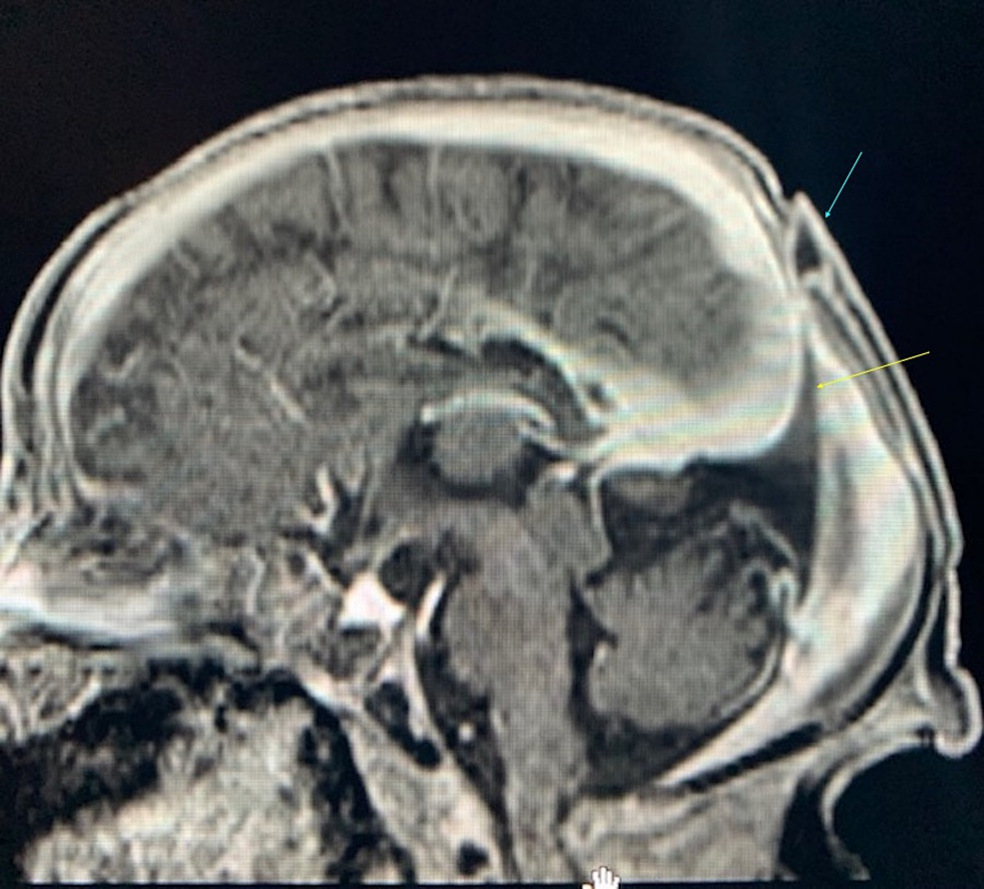
Sagittal FLAIR post-contrast MRI. The image is showing lack of enhancement at the level of scalp lesion (blue arrow), indicating a fibrous rather than venous substance, contrasting with sinus pericranii. Beaked tentorium is also shown again (yellow arrow). FLAIR: fluid-attenuated inversion recovery

**Figure 3:**
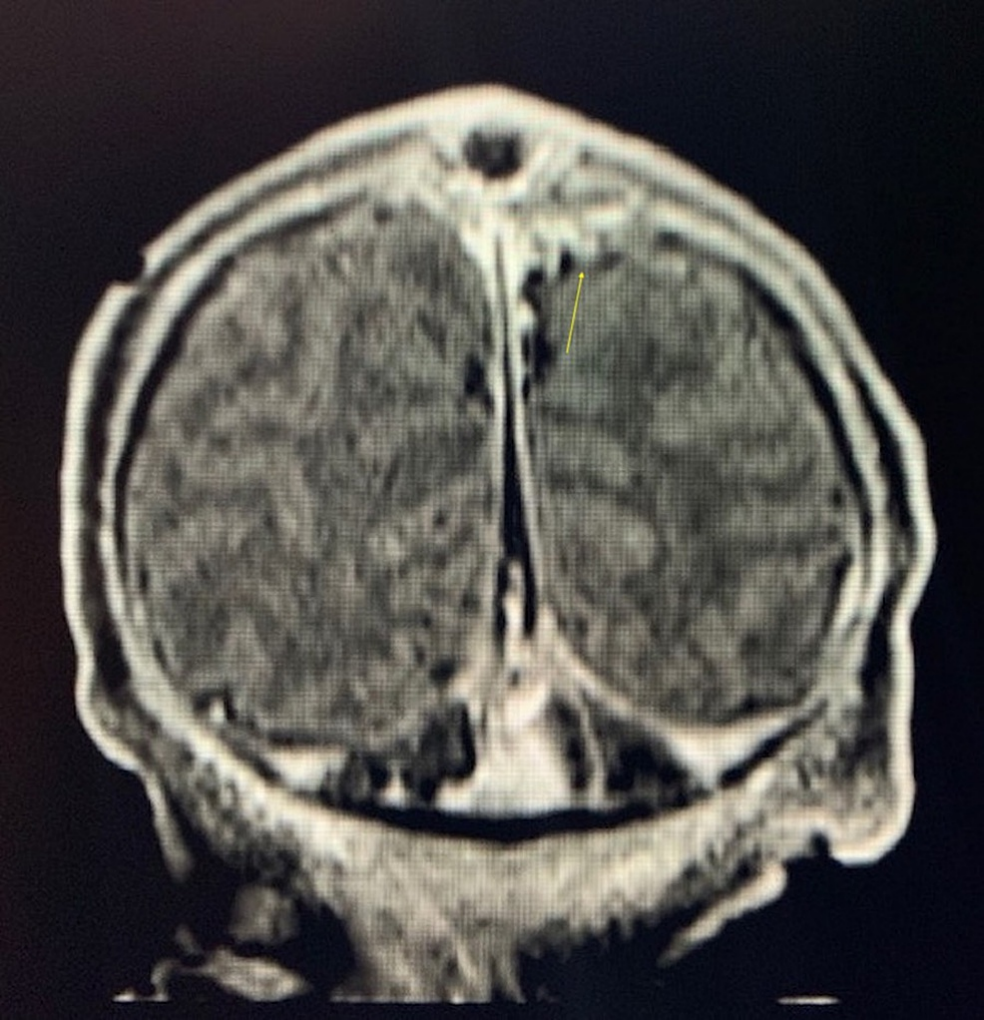
Coronal FLAIR post-contrast MRI. The image is showing fenestrations at the superior sagittal sinus on the left (yellow arrow) at the level of the cystic structure, which is a characteristic finding of APC. FLAIR: fluid-attenuated inversion recovery; APC: atretic parietal cephalocele

These findings, in addition to focal fenestration of the superior sagittal sinus at the level of the cystic structure, were compatible with an atretic parietal cephalocele (Figures 2, 3). There was also thinning of the body and splenium of the corpus callosum along with increased prominence of the cingulate sulcus, focal atrophy of the left side of the body of the corpus callosum, and mild dysplasia of the bilateral cingulate gyrus (Figure 1). No hydrocephalus was noted; however, a slight parallel configuration to the ventricles posteriorly was noted. The remainder of the MRI was normal.

## Discussion

Cephaloceles are classified by the location of extracranial dural herniation through a calvarial defect. They have often been explained as a consequence of partial dysgenesis of the neural tube [6,9]. During the development of this cyst, arachnoid cells are believed to occasionally follow the dura. Solid components often found within these cysts contain fibrocollagenous material along with atretic arachnoid cells. This quality gives certain cephaloceles the descriptor of “atretic” [10]. Clinically, this presents as a well-marginated mass located over a skull defect, in our case, over the midline parietal region. Together, these components define our case as an atretic parietal cephalocele (APC).

Atretic cephaloceles are rare, accounting for 4-17% of all cephaloceles. Of these, the parietal location accounts for 40-50% giving the overall diagnosis of APC a range of about 2-9% of all cephaloceles [11]. Diagnosis of APC after six years of age is rare, with the majority of cases presenting during the neonatal period or early childhood [12]. APC is divided pathologically into distinct categories. Type I cephaloceles contain a fibrous stalk consisting of atretic arachnoid cells surrounded by a cluster of anomalous blood vessels all contained within a dome of dermal tissues with normal hair follicles. Type II cephaloceles contain the same components with the addition of various neural/glial foci and anomalous blood vessels that extend upwards towards the dome of the lesion [9]. The vascular component of these lesions hints at an embryogenic association between APCs and various intracranial vascular defects. The most common group of anomalies associated with parietal cephaloceles consists of venous malformations and malpositioning defects [7]. These defects include vertically oriented primitive straight sinus, vertically oriented primitive falcine vein, and superior beaking of the posterior tentorium [9]. Though associations have been established between this lesion and various vascular intracranial anomalies, the origin of both remains unknown and requires further research to understand completely.

The venous channels that form during fetal development gradually coalesce to create larger channels within the meningeal layers of the embryonic neural tube [13]. Increased vascular development during various periods of fetal brain growth causes an increase in venous drainage, expanding the channels into what eventually become the dural sinuses [14]. This relationship between the sinuses and the layers of the neural tube reveals how defects in one may affect the development of the other.

**Figure 4:**
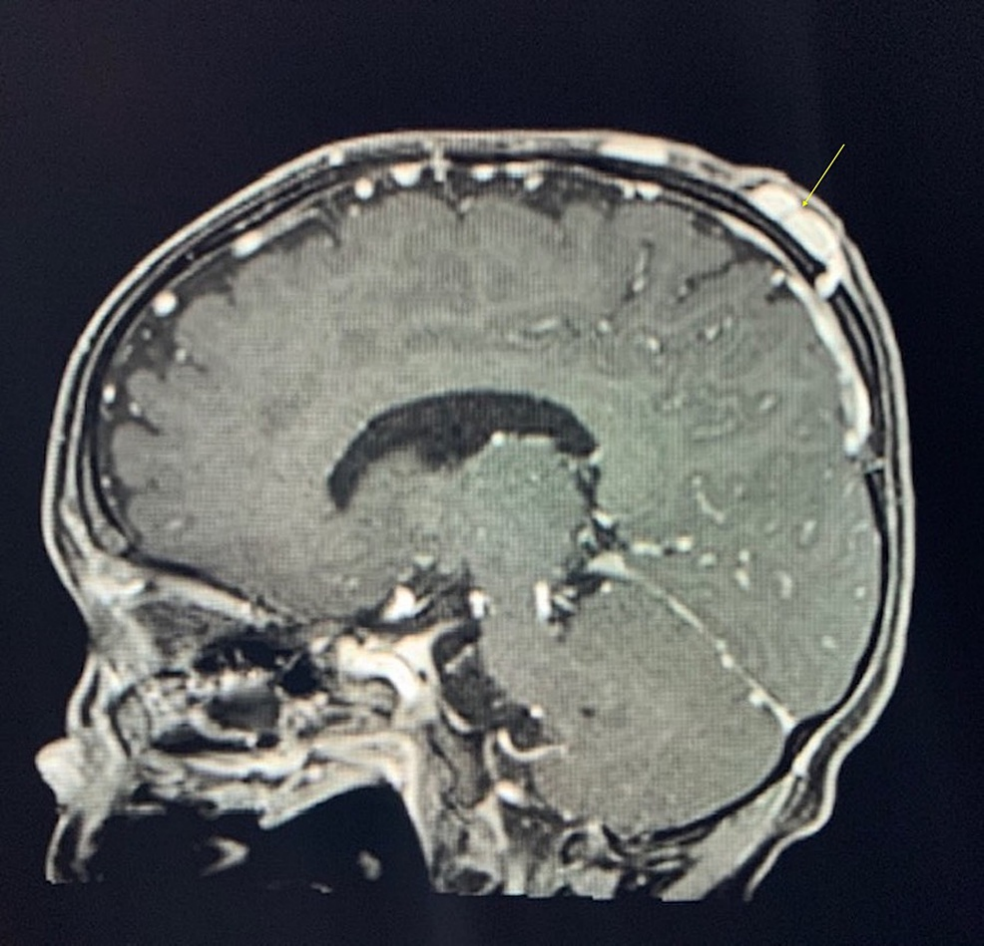
Sagittal T1 post-contrast of sinus pericranii. The image is showing enhancement of the lesions at the apex of the skull (yellow arrow) in stark comparison to weakened signal intensity seen in APC post-contrast. APC: atretic parietal encephaloceles

**Figure 5:**
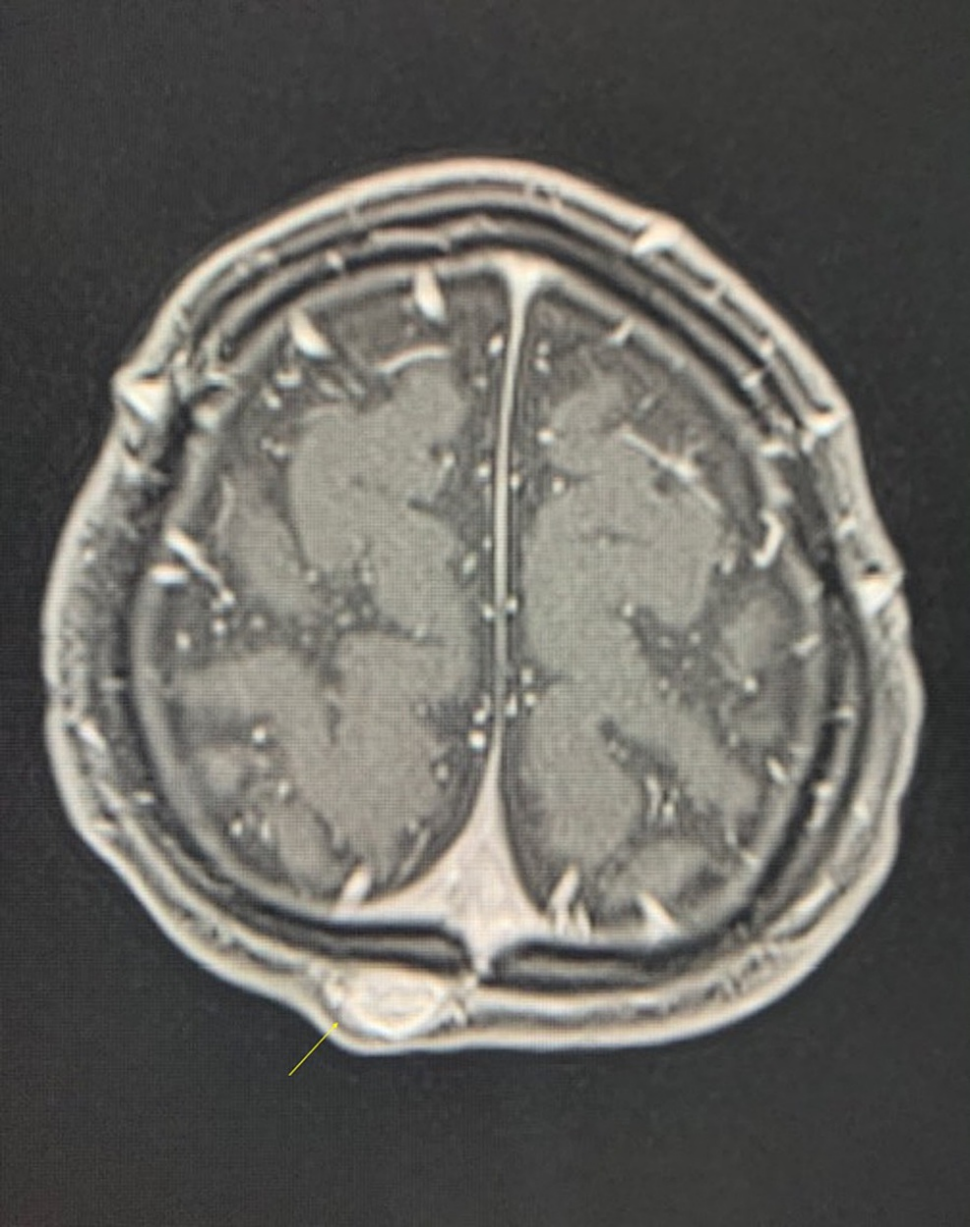
Axial T1 post-contrast of sinus pericranii. The image is showing dilated scalp vein that enhances with contrast (yellow arrow) in stark comparison to APCs. APCs do not enhance due to their fibrous nature. APC: atretic parietal encephaloceles

This association between cranial venous defects and scalp masses is evident when considering the differential diagnosis of sinus pericranii (SP) often associated with APC [9]. SP is a cranial venous anomaly that occurs when there is an irregular communication between the intracranial dural venous sinuses and extracranial venous structures, usually through an intraosseous emissary vein. This venous communication results in an external mass near the location of the communication. Like APC, this mass is most commonly midline, but it can often be distinguished clinically due to gravity-dependent filling and emptying of the SP and its fluctuant nature. Conversely, CT imaging of an SP shows thinning rather than a complete defect of the calvarial inner table. Furthermore, venous content of SP will enhance with T1-weighted MR imaging unlike APCs, which are fibrous in nature (Figures 2, 4, 5) [15].

Though diagnostic imaging can help differentiate the two, interestingly, SP and APC can occur together and are believed to be associated [16]. As aforementioned, this highlights yet another association between APC and cranial venous defects.

A common diagnostic sign of APCs is fenestration of the sagittal sinus underneath the scalp lesion. This fenestration correlates with the CSF tract seen leading to the cephalocele and its fibrous stalk [7]. Oftentimes the fibrous stalk may adhere to the vertical straight sinus at the posterior tentorium. These features are seen in our patient and they are believed to have implications regarding the embryology of this defect. Together, this can provide a timeline for when the initial insult occurred and expand upon a potential association between APCs and SPs. 

**Figure 6:**
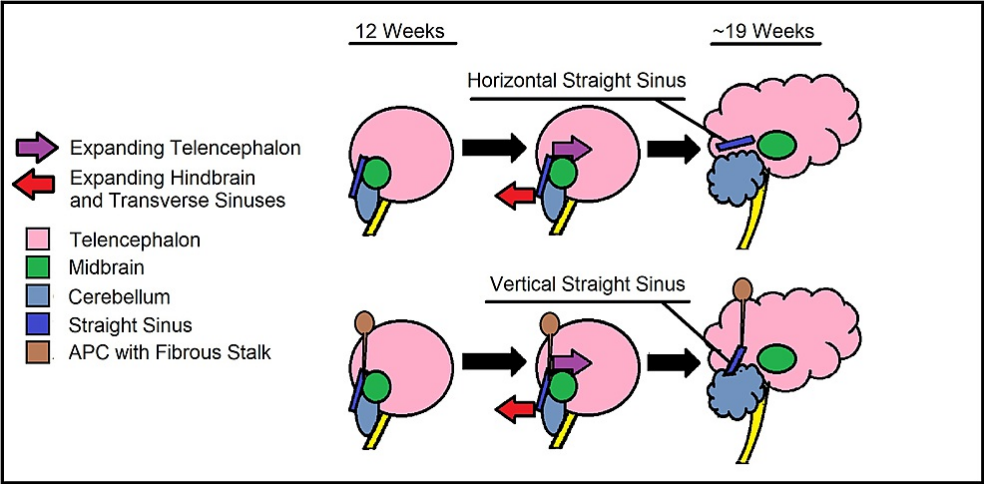
Embryology of the transverse straight sinus. Vertical straight sinus is pathological and seen only in APC, as the fibrous stalk will impede its development. APC: atretic parietal encephaloceles

During the 11th and 12th weeks of fetal development, the straight sinus can be observed in its vertical orientation. From here, the transverse sinus continues to enlarge. By the 19th week, the mass effect of the telencephalon combined with transverse sinus enlargement displaces the meningeal layers further into the developing hindbrain, causing gradual descent of the straight sinus into its adult horizontal orientation (Figure 6) [14]. In APCs, the fibrous stalk connecting to the straight sinus may prevent it from descending despite an enlarging telencephalon. Superior beaking of the posterior tentorium at the junction of the vertical straight sinus may indicate that its descent was resisted by fibrous stalk attachment (Figure 6).

This theory establishes that the fibrous stalk must have formed before the 19th week of fetal development, possibly the 11th or 12th week, when the straight sinus is first observed in its vertical orientation [7]. Additionally, traction from the fibrous stalk may also affect development of extracranial emissary veins. Potential for a communication with the superior sagittal sinus may occur, further contributing to the association between APC and SP [16].

Of note, in our patient, an occipital encephalocele and a previously described Chiari malformation were both seen during a 20-week prenatal ultrasound evaluation, both of which regressed upon follow-up. This course of apparent malformation and regression may be explained by the orientation of the straight sinus and the hypoechoic space created by its venous content. This may represent an additional consideration when assessing fetal growth via ultrasound.

In our patient, we see a cystic structure in the midline parietal region of the scalp with a connecting CSF tract. The cystic structure was identified as an APC due to its midline parietal location and the characteristic venous findings of vertically oriented straight sinus, vertically oriented primitive falcine vein, and superior beaking of the posterior tentorium [9]. Each of these findings has implications in the possible pathophysiology behind the formation of this anomaly and exploring them in tandem can help elucidate mechanisms of formation. For this reason, the clinical signs of a well-marginated midline parietal scalp mass deserve extensive imaging for pre-surgical evaluation. To summarize, APC is often the “tip of the iceberg,” hiding various anatomical surgical considerations underneath.

## Conclusions

Encephaloceles arise from a defect in the skull that allows for meningeal herniation that may or may not contain neural tissue. The etiology is not well understood, though it is regarded as a post-neurulation defect. Encephaloceles can be classified by location (frontal, occipital, or parietal), with the least common presentation being parietal. APCs are benign lesions that arise in the parietal midline. They occur due to partial neural tube closure failure, and they arise from meningeal and vestigial tissue following fibrotic degradation. In our case, we have a patient who presented with a midline parietal scalp lesion, vertically oriented straight sinus, and primitive falcine vein along with superior beaking of the tentorium, all characteristic signs of APCs. Our patient also presented with occipital encephalocele and Chiari malformation, which were both seen at a 20-week prenatal ultrasound, though this ultimately regressed. Although further investigation is required, the findings of this case may shed light on the development of APCs and relationships between origin of the defects and characteristic venous findings commonly seen in APCs. The information gathered may aid in early clinical diagnosis that can give clinicians the ability to make decisions regarding treatment of these defects early on in prenatal care. In cases such as these, further imaging studies may also allow for the prompt delivery of advanced medical management from neurosurgery teams and other specialists that may improve morbidity and mortality among these types of defects.
